# Idiopathic Transient Osteoporosis of the Hip: A Frequently Overlooked and Misdiagnosed Cause of Hip Pain

**DOI:** 10.7759/cureus.75881

**Published:** 2024-12-17

**Authors:** Ana Flávia Resende, Margarida Teixeira, Zico Gonçalves, Eduardo Mendes, Francisco Agostinho

**Affiliations:** 1 Orthopedics and Traumatology, Unidade Local de Saúde de Viseu Dão-Lafões, Viseu, PRT; 2 Orthopedics and Traumatology, CUF Viseu Hospital, Viseu, PRT

**Keywords:** case reports, conservative treatment, hip, pain management, transient osteoporosis

## Abstract

Idiopathic transient osteoporosis of the hip (ITOH) is a rare and self-limiting condition of unknown origin, typically responding well to conservative treatment. It is characterized by progressive pain, claudication, and osteoporosis of the femoral head, while the joint line remains preserved. Early clinical and radiological findings can be misinterpreted as aseptic necrosis, infection, or neoplastic processes, making careful exclusion of these conditions essential. In this report, we describe the case of a 55-year-old male presenting with left-sided hip pain without any preceding trauma. After ruling out other potential conditions, ITOH was suspected, and the patient was successfully managed conservatively. ITOH continues to be a poorly understood and frequently overlooked clinical entity. Despite advancements in imaging techniques, its diagnosis remains challenging and often depends on exclusion. This case highlights the importance of recognizing and discussing ITOH in clinical practice.

## Introduction

Idiopathic transient osteoporosis of the hip (ITOH) is a rare, self-limiting condition characterized by unknown etiology and generally of benign clinical progression [1]. The variability in nomenclature observed in the literature highlights a significant gap in understanding and emphasizes the importance of establishing standardized diagnostic criteria [2]. 

The hip joint is most commonly affected, though other joints, including the knee, ankle, shoulder, elbow, wrist, and smaller joints, may also be involved. This condition typically affects middle-aged men and women, particularly during the third trimester of pregnancy or the postpartum period, though cases in non-pregnant women have also been documented [3]. 

Clinical suspicion is primarily guided by patient history and physical examination findings, while MRI plays a crucial role in confirming the diagnosis and excluding differential conditions [3,4].

The delay in the diagnosis process, compounded by limited awareness of this poorly understood and frequently overlooked entity, can result in inappropriate treatment [4]. Thus study aims to present a clinical case and to emphasize the significance of considering ITOH as a differential diagnosis in patients presenting with hip pain.

## Case presentation

A 55-year-old male patient presented to the outpatient clinic with progressive left hip pain of non-traumatic origin, persisting for three months with a mechanical pattern. The patient denied experiencing fever or systemic symptoms such as fatigue, weight loss, or anorexia. He had previously sought medical attention for the same complaint and was prescribed analgesics, which provided limited relief.

His medical history was unremarkable, and he reported no regular medication use or alcohol or tobacco consumption.

On physical examination, the patient exhibited pain during hip flexion and rotational movements, with no other notable findings. There were no motor or neurological deficits.

The diagnostic workup included laboratory investigations, which showed normal levels of inflammatory markers, thyroid function, uric acid, rheumatologic markers, and calcium metabolism.

**Figure 1:**
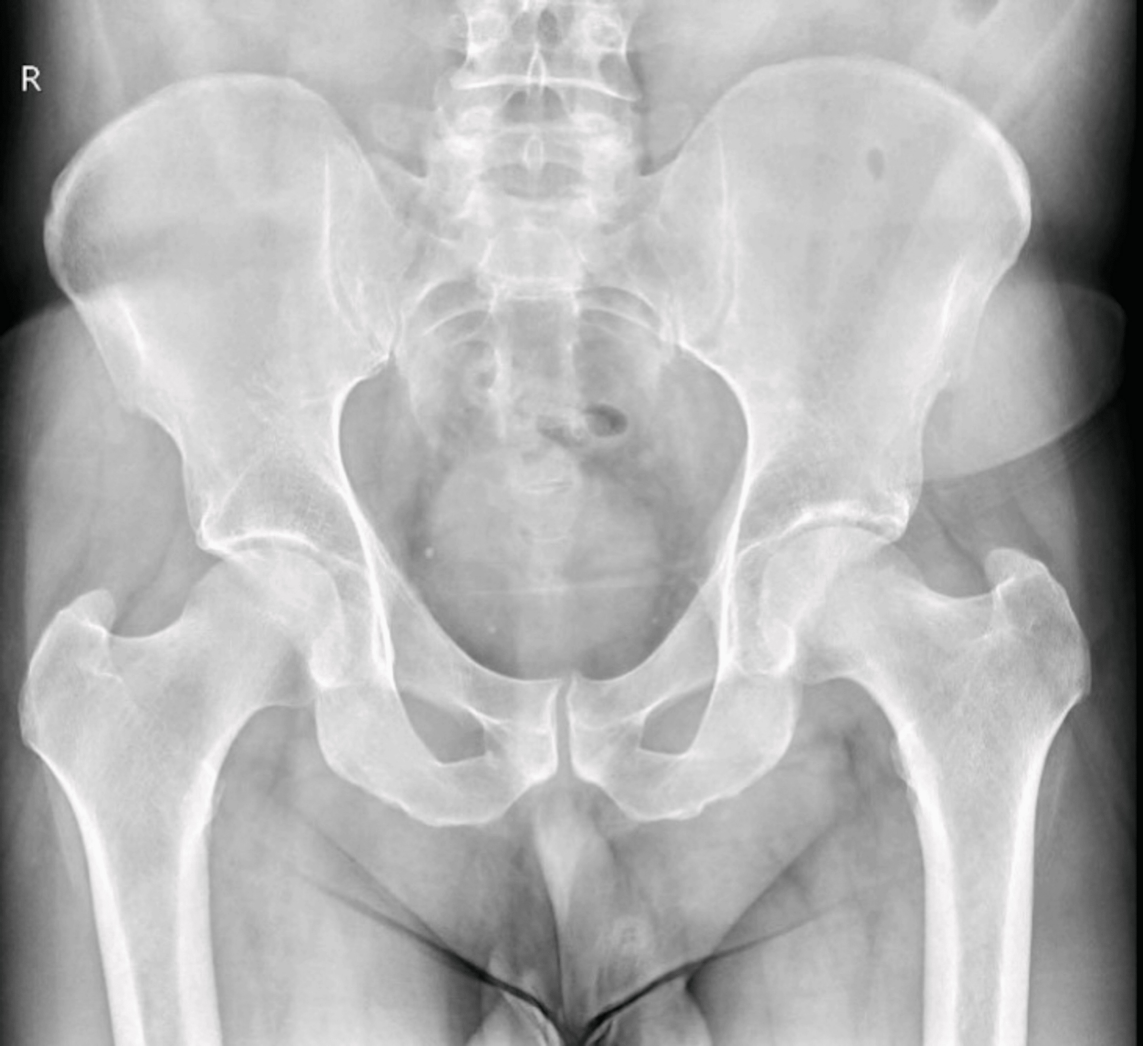
Anteroposterior radiograph of the pelvis showing no acute abnormalities at symptom onset

**Figure 2:**
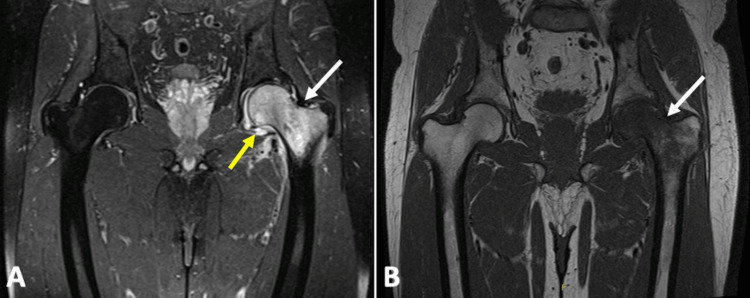
MRI scan performed after three months of symptoms On T2-weighted images(A), hyperintense signal is noted in the proximal femur (white arrow), along with evident joint effusion (yellow arrow). Hypointense signal is observed on T1-weighted images (B) in the same location, with preservation of the joint space.

Plain radiographs of the hip and pelvis (Figure 1) showed no apparent abnormalities. However, MRI identified edema of the left femoral head extending into the intertrochanteric region, with a linear subchondral hypointense signal in the superior portion of the femoral head. There was significant joint effusion and edematous infiltration of periarticular soft tissues, with no acetabular abnormalities noted (Figure 2). 

After excluding more probable differential diagnoses, ITOH was considered the most likely diagnosis. The patient was treated conservatively with partial weight-bearing on the affected limb and analgesics.

At six months following the diagnosis, the patient exhibited complete clinical recovery, reporting no symptoms or complications. His functional assessment using the Harris Hip Score yielded a score of 93, indicating excellent hip function. Additionally, he successfully resumed recreational sports activities without experiencing any discomfort or limitations. 

## Discussion

ITOH is a rare and benign clinical entity first described in 1959 by Curtis and Kincaid [5]. It is characterized by a transient episode of bone marrow edema, as observed through MRI, accompanied by hip pain of unknown cause and functional limitation [6]. Several terms have been used in the literature to describe the condition of transient bone marrow edema, including regional migratory osteoporosis, transient bone marrow edema syndrome, reflex sympathetic dystrophy, and transient idiopathic osteoporosis. The various terms used to describe this condition reflect not only the clinical and radiological characteristics but also the lack of understanding and the challenges involved in diagnosing this pathology [6,7]. Therefore, it is essential to differentiate ITOH from other causes of transient bone marrow edema in the hip, including inflammatory, degenerative, neoplastic, or vascular origins [6]. 

For this condition, several risk factors have been described in the literature, such as corticosteroid use, smoking, hypothyroidism, decreased testosterone levels, vitamin D deficiency, pregnancy, and breastfeeding, among others [1,6]. However, the causal relationship between these factors and the development of ITOH has not been firmly established in the literature [1,6,8].

Given its rarity, non-specific symptoms, and unpredictable episodes, ITOH is often misdiagnosed [9,10].

According to some studides, the condition can be clinically and radiologically defined in three phases [3,7,11,12]. Phase 1 is where the patient presents with pain, walking difficulties, and possible limp. Radiographs show no abnormalities, while MRI reveals diffuse bone marrow edema. Phase 2 is where pain begins to decrease, and radiographs show hypodensity with maintenance of the joint line. MRI reveals lesions with hypointensity on T1-weighted images and hyperintensity on T2-weighted images, without collapse of the femoral head. Phase 3 refers to the resolution of the condition, with improvement in pain symptoms and healing of bone lesions in the subsequent months. 

Analytical study typically shows no abnormalities, as there are no specific biomarkers or tests to aid in the diagnosis of this pathology. Therefore, its primary role its primary role is to exclude more aggressive conditions, such as metabolic or metastatic diseases [8].

In its early stages, this condition can be easily mistaken for other disorders. Consequently, careful monitoring of the initial symptoms and their progression is crucial to avoid unnecessary procedures and treatments [1,8,13].

Differential diagnoses include osteomyelitis, septic or inflammatory arthritis, neoplasia, stress fractures, and avascular necrosis (AVN) [4,8,13].

Distinguishing between ITOH and AVN can be challenging, particularly in the early stages of AVN, where both radiological and clinical findings may appear identical. Clinically, pain associated with ITOH typically improves with rest and weight-bearing activities. In contrast, AVN is characterized by progressive, insidious pain that does not alleviate with weight-bearing and is also associated with a significant reduction in the range of motion. Radiologically, it may present with femoral head deformity, subchondral radiolucency (crescent sign), and indirect signs of joint edema [4,13,14].

Inflammatory or septic conditions typically present with warmth, erythema, fever, swelling, and restricted movement, with positive findings on joint aspiration and elevated inflammatory markers, whereas ITOH lacks signs of infection and inflammatory systemic symptoms [4,8].

Neoplasia may be suspected if there are persistent, unexplained symptoms or abnormal imaging findings, such as bone masses. Stress fractures, unlike ITOH, generally result from repetitive mechanical stress and are often identified through imaging showing bone discontinuity [3,4,8].

ITOH is a benign condition, and as previously mentioned, it is self-limiting. There is a significant loss of trabecular architecture, which leads to a decline in local bone strength. Although the edema resolves, the mechanical strength remains diminished. Therefore, the goal of treatment is not only to manage pain but also to provide partial protective weight-bearing until until bone mineral density is restored [1,8].

Various pharmacological treatments have been proposed for managing the condition, including non-steroidal anti-inflammatory drugs (NSAIDs), bisphosphonates, calcitonin, teriparatide, iloprost [1,8,9]. Additionally, non-pharmacological interventions, such as hyperbaric oxygen therapy (HBOT) and extracorporeal shockwave therapy (ESWT), have also been described [1,9,14]. For patients who do not respond to conservative management, core decompression (CD) remains the surgical treatment of election [14,15].

Bisphosphonates and calcitonin exhibit anti-inflammatory properties by inhibiting osteoclastic activity and bone resorption. Teriparatide promotes osteoblastic activity [1]. Iloprost, a synthetic prostacyclin (PGI2) analog, induces vasodilation, promotes interstitial fluid absorption, reduces bone pressure and it may also stimulate osteoblastic activity [14]. HBOT enhances bone metabolism, collagen production, fibroblast proliferation, and capillary formation through increased oxygenation, while its vasoactive effects reduce edema and improve joint microcirculation [15]. ESWT promotes angiogenesis, activates osteoblasts and periosteal cells, and induces osteogenic differentiation of mesenchymal stem cells, enhancing bone deposition and remodeling by reducing pro-osteoclastogenic factors [14].

There are no established guidelines for treatment options, and the majority of the literature consists of case series or retrospective cohort studies, which provide a low level of evidence [1,2,10,14].

In a recent meta-analysis, Paraskevopoulos et al. compared the various treatment modalitiesfor the management of this condition and showed that the effectiveness of treatments (bisphosphonates, ilioprost, CD and NSAIDS/analgesics/weigh bearing) for pain and MRI resolution varied by timeline and modality [14]. Bisphosphonates showed best outcomes at early stage (within one month), iloprost at mid-term (three months), and ESWT and CD showing superior results in the long term (above six months). Nevertheless, these findings are based only on observational data since there is a lack of statistical evidence to support them.

Due to the paucity of studies comparing surgical intervention to other treatment options, it is challenging to determine when a patient requires surgery after conservative management fails [1,10,14].

Further studies are needed to clarify effects of pharmacological and non-pharmacological treatments as well as to establish routine recommendations for their use [2,10,14].

Subchondral fractures, femoral neck fractures, and subcapital fractures, though rare, are a potential complication of ITOH. The incidence of subchondral fractures is similar between genders, while the incidence of other fractures is higher in women. This increased incidence is thought to be related to hormonal factors associated with pregnancy and childbirth. Progression of ITOH to AVN or osteonecrosis is another rare but potential complication, making it important to remain vigilant for this possibility [10,14].

## Conclusions

Despite advances in imaging techniques, diagnosing ITOH remains challenging due to non-specific clinical and imaging findings, often overlapping with other conditions. Early and accurate differentiation is crucial, as it allows for the timely identification and treatment of other conditions with a more severe prognosis.

Current literature reports promising results regarding treatment options in reducing the clinical course. However, further studies are required, along with the establishment of standardized radiological and clinical scoring systems, to provide evidence-based recommendations for the therapeutic algorithm.
